# Predicting outcome in frozen shoulder (shoulder capsulitis) in presence of comorbidity as measured with subjective health complaints and neuroticism

**DOI:** 10.1186/s12891-017-1740-9

**Published:** 2017-09-02

**Authors:** Satya Pal Sharma, Rolf Moe-Nilssen, Alice Kvåle, Anders Bærheim

**Affiliations:** 1 Research Group, Section for General Practice, Department of Global health and Primary care, University of Bergen, Kalfarveien 31, N-5018 Bergen, Norway; 2 Physiotherapy Research Group, Department of Global Public Health and Primary Care, University of Bergen, Bergen, Norway; 3 Department of Health and Social Sciences, Western Norway University of Applied Sciences, Bergen, Norway

**Keywords:** Frozen shoulder, Shoulder capsulitis, Shoulder pain and disability index, SPADI, Subjective health complaints, SHC, Neuroticism

## Abstract

**Background:**

There is a substantive lack of knowledge about comorbidity in patients with frozen shoulder. The aim of this study was to investigate whether subjective health complaints and Neuroticism would predict treatment outcome in patients diagnosed with frozen shoulder as measured by the Shoulder Pain and Disability Index (SPADI) and change in SPADI.

**Methods:**

A total of 105 patients with frozen shoulder were recruited for a randomised controlled trial, where 69 were in the intervention group and received intraarticular corticosteroid injections and 36 patients served as control group. The SPADI was used as the outcome measure after 8 weeks, and change in SPADI from baseline to 8 weeks as a measure of rate of recovery. To examine comorbidities, all participants completed the Subjective Health Complaints (SHC) questionnaire with its five subscales, and the Neuroticism (N) component of the Eysenck Personality Questionnaire Revised. Multiple regression analysis was performed with the baseline comorbidity variables that correlated significantly with SPADI after 8 weeks, and with change in SPADI from baseline to 8 weeks, controlling for the variables intervention, age, gender and duration of pain.

**Results:**

In this study, patients with frozen shoulder had little comorbidity as measured with SHC and scored normally with respect to Neuroticism. Only the Pseudoneurology subscale in SHC correlated significantly with SPADI and had significant predictive power (*p* < 0.001) for the outcome at 8 weeks. The intervention group exhibited significant statistical predictive power (*p* < 0.001) for the treatment outcome as measured by a change in SPADI from baseline to 8 weeks. Being female also had some predictive significance for change in SPADI (*p* < 0.005).

**Conclusion:**

Psychometric parameters as measured by the Pseudoneurology subscale in SHC questionnaire did predict the treatment outcome in frozen shoulder as measured by SPADI at 8 weeks, but not by change in SPADI from baseline to 8 weeks. One may conclude that psychometric parameters may affect symptoms, but do not predict the rate of recovery in frozen shoulder.

**Trial registration:**

ClinicalTrials.gov, identifier: NCT01570985.

## Background

Frozen shoulder or capsulitis of the shoulder has a prevalence of 2–5% in the general population and occurs mostly in middle age between 40 and 60 years. Women are more commonly affected than men [1–4]. Both shoulders can be affected simultaneously or one side becomes affected first and then the other side a few years later in 6–17% of patients [5–7]. One has observed a significantly adverse impact on pain, function and quality of life in patients with shoulder adhesive capsulitis as measured with Shoulder Pain and Disability Index (SPADI) and the Short Form survey-36 (SF-36) [8]. The burden of shoulder conditions, in terms of affecting a patient’s perception of his or her general health, has been ranked as highly as the burden of having any of hypertension, congestive heart failure, acute myocardial infarction, diabetes mellitus and/or depression [9, 10]. In a systematic review of prognostic factors for arm, neck and shoulder complaints, the duration and degree of symptoms and resulting limitation of shoulder function were prognostic for recovery [11]. Another systematic review found that a high SPADI score [12], in addition to greater severity and longer duration of shoulder pain were associated with becoming the shoulder pain chronic [13]. Kuijpers et al. had similar findings regarding the duration and severity of pain at the time of presentation and its association with chronic shoulder pain [14]. Comorbid factors had a significant effect on pain and dysfunction, as measured on shoulder-specific and general health instruments, experienced by patients with adhesive shoulder capsulitis [15]. General health may also be seen to improve after reduction in shoulder pain and dysfunction. Functional outcome as measured by SF-36 after arthroscopic release in refractory adhesive shoulder capsulitis, improved clinical and general health status for most of the patients [16, 17].

Several questionnaires are available to measure complaints among patients with chronic conditions [18]. In Nordic countries, a validated questionnaire consisting of 29 parameters have been used to measure severity and duration of subjective somatic and psychological complaints during the previous 30 days. The SHC questionnaire is a systematic, easy and reliable way to measure subjective health status and comorbidity [19]. It is also argued, that what may be termed as “medically unexplained symptoms” or functional somatic syndromes [20], are better covered under “subjective health complaints” [21]. Subjective health complaints concerning musculoskeletal disorders, the digestive system, tiredness, dizziness, sleep and unspecific pain etc. are common in the general population [22, 23]. Prevalence of reported SHC was found high in general Norwegian population, where 80% reported diverse musculoskeletal complaints e.g. headache, neck pain, back pain, pain in the arms, shoulder pain, migraine and or pain in the feet on exertion. Whereas 65% reported “pseudoneurological” complaints, including sleep problems, tiredness, anxiety, depression, dizziness, hot flushes and/or extra systoles, among others [23]. The SHC questionnaire has not been used earlier for measuring health status in patients with frozen shoulder.

Apart from the clinical characteristics, psychological factors also play a role in predicting outcome of neck and shoulder symptoms [24]. The relationship between psycho-social factors particularly related to work environment and development of musculoskeletal complaints and the transition to a chronic state has been hypothesized by some authors and explanatory models are suggested [25, 26]. Further, the physical and psycho-social disability in patients with chronic pain has been shown to be associated with patients’ pain-related beliefs [27]. Pain related fear and avoidance is postulated to be an essential feature of chronification of pain for at least some patients [28].

Neuroticism is a broad personality trait that reflects the extent to which a person experiences the world as stressful, threatening, and problematic. “Neuroticism has been linked to a wide array of clinical syndromes, with particularly strong associations to distress-based disorders such as major depression and generalized anxiety disorder. The trait has also been found to be a significant predictor of Subjective Health Complaints” [29]. The aspect that whether patients with frozen shoulder have comorbidity and have neurotic symptoms that may influence response to treatment has previously not been studied.

### Aim

The aim of this study was to investigate whether Subjective Health Complaints and Neuroticism would predict treatment outcome in patients diagnosed with frozen shoulder as measured by SPADI and change in SPADI.

### Hypothesis

Comorbidity as measured with SHC and Neuroticism at baseline can predict outcome in frozen shoulder as measured by SPADI at 8 weeks and change in SPADI from baseline to 8 weeks.

## Methods

Patients in this study were participants in a randomised controlled trial (RCT), where 69 were in the intervention group and received intraarticular corticosteroid injections during a period of 8 weeks and 36 patients were in the control group [30]. Most of the patients were in stage II of frozen shoulder with SPADI score around 60. The SPADI was used as the outcome measure. We measured SPADI at 8 weeks and change in SPADI from baseline to 8 weeks. We interpret change in SPADI as a measure of rate of recovery. There were statistically significant differences between those receiving intervention and the control group after 8 weeks in the primary outcome measure. At inclusion and after 8 weeks, comorbidity was measured with the SHC questionnaire and Neuroticism was measured with the Neuroticism (N) component of the Norwegian version of the Eysenck Personality Questionnaire- Revised short form (EPQ-R) [31–33].

SPADI measures a combination of pain and functional disability on a score ranging from 0 to 100, a high total score indicating more pain and disability [12]. In the SHC questionnaire, severity of each complaint is rated on a 4-point scale (0 = none, 1 = some, 2 = much, 3 = severe). In this study, we calculated the total SHC scores for 29 items and differentiated SHC scores in five subscales. These are: Musculoskeletal (comprising headache, neck pain, back pain, pain in arms, shoulder pain, migraine and pain in feet on exertion); Pseudoneurology (sleep problems, tiredness, anxiety, depression, dizziness, hot flushes and extra systoles); Gastrointestinal (stomach discomfort, heartburn, ulcer/non-ulcer dyspepsia, stomach pain, flatulence, diarrhea and obstipation), Cold/Flu (flu, bad cold, cough/bronchitis) and Allergy (asthma, chest pain, breathing difficulty, eczema and allergy) [19]. The EPQ-R questionnaire has four scales: E (Extraversion vs. Introversion), N (Neuroticism or Emotionality), P (Psychoticism or Tough Mindedness) and L (Lie scale). The short form Neuroticism (N) questionnaire has 12 questions to be answered with yes or no options and only this part was used in this study [31].

### Statistics

Baseline variables of comorbidity and Neuroticism were explored. To have an overview of the burden of symptoms, we performed descriptive analysis of SHC with its subscales and Neuroticism. To select appropriate baseline scores as predictors of outcome, we explored correlations between SPADI at 8 weeks and change in SPADI from baseline to 8 weeks with baseline SHC total and subscale scores, and with the Neuroticism sum score. Multiple regression analysis was performed with the items that correlated significantly with SPADI at 8 weeks and change in SPADI from baseline to 8 weeks as predictors, controlling for intervention, age, gender and duration of shoulder pain. We chose a backward elimination method for multiple regression analysis, and removed non-significant predictors one by one. Both initial and final models are reported.

## Results

Baseline characteristics of patients and SPADI are displayed in Tables 1 and 2. There were no noteworthy differences in the demography between the intervention and control groups at baseline except for a higher percentage of patients with trauma in the intervention group. The baseline SPADI was similar in the two groups (Table 2). Descriptive scores for SHC and for Neuroticism at baseline and 8 weeks presented in Table 3, show relatively low prevalence of health complaints. The preliminary correlation analysis returned significant Pearson’s correlation coefficients for SPADI at 8 weeks versus total SHC score at baseline, the Pseudoneurology subscale in SHC at baseline (*p* = 0.009), as well as group allocation (*p* < 0.001) (Table 4). None of the other SHC subscales at baseline returned significant correlation coefficients. There was a significant correlation between female gender and SPADI at baseline. When we removed the Pseudoneurology subscale from the total SHC score at baseline, the remaining total SHC score became insignificant. This showed that the significant correlation coefficient related to the total SHC baseline score was due to inclusion of the Pseudoneurology subscale. Therefore, only the Pseudoneurology subscale and not the total SHC baseline score was kept as predictor in the multiple regression analysis. No correlation was found between baseline Neuroticism and the outcome measure after 8 weeks. Correlation analysis with change in SPADI from baseline to 8 weeks showed statistically significant correlation to group allocation and female gender (Table 4).

**Table 1:** Baseline characteristics of patients diagnosed with frozen shoulder

Characteristics	Intervention group Number and % within group *n* = 69	Control group Number and % within group *n* = 36
Mean age (years)	53 (8.7)	54 (6.9)
Female	42 (60%)	19 (53%)
Duration in months: Median (range)	7.2 (2.0–37.0)	6.0 (3.0–24.0)
Affected right shoulder	30 (43%)	15 (42%)
Previous frozen shoulder	10 (14%)	4 (11%)
Concurrent neck pain	31 (44%)	16 (44%)
Trauma to shoulder	13 (19%)	3 (8%)
Previous operation on shoulder	6 (9%)	1 (3%)
Dominant right side	64 (91%)	34 (94%)
Previous shoulder treatment	37 (53%)	13 (36%)
Analgesics	33 (47%)	11 (31%)
Participants on sick leave	14 (20%)	15 (42%)
Smokers	6 (18%)	12 (33%)

**Table 2:** SPADI at baseline and 8 weeks, and change in SPADI from baseline to 8 weeks for the intervention and control groups

SPADI	Intervention group Mean, (Std. deviation)	Control group Mean, (Std. deviation)
Baseline	62.3 (16.4)	61.4 (19.07)
8 weeks	22.2 (20.3)	43.5 (23.8)
Change from baseline to 8 weeks	40.2 (19.0)	17.8 (15.0)

**Table 3:** Subjective Health Complaints (SHC) and Neuroticism in the intervention and the control group at baseline and 8 weeks

	Intervention group Mean, (Std. deviation)		Control group Mean, (Std. deviation)	
	Baseline	8 weeks	Baseline	8 weeks
SHC - Total score 29 items (score 0–87)	15.34 (8.16)	11.33 (8.04)	12.22 (6.66)	11.08 (7.98)
Musculoskeletal (8 items) (score 0–24)	8.11 (4.06)	5.62 (3.87)	7.44 (3.45)	6.50 (3.57)
Pseudoneurology (7 items) (score 0–21)	4.17 (3.03)	3.23 (2.98)	3.11 (2.67)	2.86 (3.07)
Gastrointestinal (7 items) (score 0–21)	1.70 (2.30)	1.35 (1.92)	0.92 (1.44)	0.94 (1.66)
Flu (2 items) (score 0–9)	0.57 (1.11)	0.67 (1.07)	0.56 (1.27)	0.53 (1.08)
Allergy (5 items) (score 0–15)	0.76 (1.36)	0.48 (0.95)	0.25 (0.55)	0.36 (1.05)
Neuroticism (score 0–12)	2.42 (2.14)	1.57 (2.05)	2.06 (2.28)	1.50 (1.80)

**Table 4:** Pearson’s correlations^a^ between independent variables and SPADI at 8 weeks and change in SPADI from baseline to 8 weeks

Correlated independent variables	SPADI at 8 weeks		Change in SPADI (baseline to 8 weeks)	
	Pearson’s r	*p*-value	Pearson’s r	*p*-value
SHC Pseudoneurology baseline	0.26^*^	0.009	0.11	0.287
Gender (male = 0; female = 1)	0.08	0.429	0.26^*^	0.007
Group allocation (control = 0; intervention = 1)	− 0.45^*^	< 0.001	− 0.43^*^	< 0.001
Shoulder pain duration	− 0.16	0.108	0.28^*^	0.004

^*^ *p* < 0.05

^a^Only variables with significant correlations are listed in the table

Multiple regression analysis with SPADI at 8 weeks as the dependent variable, controlling for age and gender, revealed a statistically significant predictive value for Pseudoneurology in SHC at baseline (*p* < 0.001) and group allocation (*p* < 0.001) (Table 5). Shoulder pain duration was also registered but did not show any statistically significant predictive value.

**Table 5:** Multiple regression models with backward elimination for participants (*n* = 105, control and intervention groups) with the Shoulder Pain and Disability Index (SPADI) at 8 weeks and change in SPADI from baseline to 8 weeks as dependent variables

	Initial model			Final model		
	B	*p*-value	R^2^	B	*p*-value	R^2^
SPADI at 8 weeks			0.30			0.29
SHC Pseudoneurology baseline	2.4	0.001		2.6	< 0.001	
Gender (male = 0; female = 1)	0.78	0.85				
Age	− 0.36	0.15				
Group allocation (control = 0; intervention = 1)	− 25.1	< 0.001		− 24.8	< 0.001	
Change in SPADI from baseline to 8 weeks			0.36			0.36
SHC Pseudoneurology baseline	0.19	0.77				
Gender (male = 0; female = 1)	11.78	0.002		11.99	0.002	
Age	0.48	0.04		0.47	0.04	
Group allocation (control = 0; intervention = 1)	19.50	< 0.001		19.73	< 0.001	
Shoulder pain duration	0.93			0.91	0.013	

Being allocated to the intervention or control group exhibited statistical significant predictive value. This was also the case for change in SPADI from baseline to 8 weeks (*p* < 0.001). Baseline SHC scores did not have significant predictive value at 8 weeks for a change in SPADI. Shoulder pain duration showed a statistically significant predictive value for change in SPADI from baseline to 8 weeks (*p* < 0.01).

## Discussion

In this study, we found that patients with frozen shoulder had little comorbidity as measured with SHC and they scored normally on the Neuroticism questionnaire. We found that both the SHC Pseudoneurology subscale and group allocation predicted pain and function as measured by SPADI at 8 weeks. However, when looking at factors predicting change in SPADI from baseline to 8 weeks, shoulder pain duration and group allocation predicted better outcome, while SHC as a whole and each subscale lost its predictive power.

Most of the participants were in stage II of frozen shoulder [30] with relatively high baseline SPADI score (Table 2). The mean duration of frozen shoulder at the time of inclusion in the study was 6 months (Table 1). Some patients go through a very painful phase and delayed diagnosis resulting in frustration, anxiety and depression. Jones et al. observed in their study that lack of diagnosis or misdiagnosis led to diverse consequences among the participants; for example, anxiety, denial and delays in definitive diagnosis and referral [34]. In patients with cervical radiculopathy, variables regarding present neck pain intensity, fear avoidance and anxiety were most significant in dimensions underlying pain and disability, personal factors and health status [35]. One of the major complaints in frozen shoulder in late stage I and stage II is pain which in some cases can be very severe [36], resulting in very disturbed sleep and tiredness. Perceived disability in patients with chronic shoulder pain has been found to be strongly influenced by depressive symptoms [37]. In our study, 44% of patients in both groups also had neck pain, which may have contributed to elevated self-experience of pain or disability (Table 1). It is possible, that after receiving information at the time of inclusion in the study regarding frozen shoulder and its natural course patient’s anxiety and depressive symptoms were reduced. The Pseudoneurology subscale was no longer significantly predictive with regard to change in SPADI from baseline to 8 weeks.

In this study, the follow up was limited to 8 weeks and this may affect external validity. A further follow up at 6 or 12 months would have been appropriate to predict long-term outcome. This is a limitation of this study.

Since we lack reference values for SHC and Neuroticism, we cannot compare our findings with the general population. This is also a limitation of the study.

In our study, Neuroticism did not have any significance in predicting the outcome of frozen shoulder. Others have found that personality factors may modulate presentation of pain and symptoms and influence a broad range of health outcomes and mechanisms [20, 38]. Rozencwaig et al. have demonstrated that the number of medical conditions has a quantitative effect on shoulder function. The parameters for general health perception and vitality in the SF-36 questionnaire has previously been found to have a strong negative correlation with the increasing comorbidity in patients with gleno-humeral degenerative joint disease [39].

Belonging to the intervention group had significant predictive value (*p* < 0.001) for both SPADI at 8 weeks and change in SPADI from baseline to 8 weeks. Contrary to what we had expected, SHC total score, SHC subscales and Neuroticism had no predictive value for change in SPADI from baseline to 8 weeks. We expected the Musculoskeletal subscale to have a predictive power because this subscale contains parameters regarding neck pain, back pain, pain in arms, shoulder pain and pain in feet, which are relevant to this study. We do not have any good explanation for this lack of predictive influence. Absence of predictive power for Neuroticism is in accordance with findings in other studies. Ring et al. found that self-reported upper extremity-specific health status correlated with the Disabilities of the Arm, Shoulder and Hand (DASH) questionnaire, but not with Neuroticism, as measured by the EPQ-R [40]. Factorial analysis of subjectively felt health complaints by Ursin et al. revealed that factors involving neck, back, arm and shoulder pain and migraine, did not relate to anxiety and depression [41]. Psychological factors explained only a moderate amount of variance of muscle pain, when the population was looked at as a whole, in their study [41]. This is similar to our findings, i.e. the Musculoskeletal subscale did not show predictive power for SPADI. In general, psychological comorbidity has been found to enhance self-experience of suffering due to pain and dysfunction. Bagheri et al. reported more suffering due to depression and anxiety than that from a reduced range of motion in patients with frozen shoulder [42]. Further, physical and psychosocial disability in patients with chronic pain have been shown to be associated with patients’ pain-related beliefs [27]. Patients with psychological disorders have been found to have more self-reported pain and functional disability in activities of daily life, indicating correlation with frozen shoulder and psychological conditions [43]. However, the Musculoskeletal subscale did not predict the outcome in our study even though it has components of pain parameters. There is a possibility that the SHC and Neuroticism questionnaires are not able to measure psychometric parameters when these are not sufficiently accentuated or are dominating the clinical picture. However, frozen shoulder may be a very distinct physical and clinical entity, without associated psychological aspects. When patients are informed of the diagnosis, its natural history and possible outcome after intervention, the condition is no longer dramatic and they cope well with it. According to available literature, psychometric parameters can in some way affect the outcome [15, 24–26], but it was not obvious or consistent in our study.

## Conclusion

Psychometric parameters as measured by the Pseudoneurology subscale in SHC questionnaire did predict the treatment outcome in frozen shoulder as measured by SPADI at 8 weeks, but not by change in SPADI from baseline to 8 weeks. One may conclude that psychometric parameters may affect symptoms, but do not predict the rate of recovery in frozen shoulder.

## Abbreviations

EPQ-R: Eysenck personality questionnaire- revised
SF-36: Short form survey-36
SHC: Subjective health complaints
SPADI: Shoulder pain and disability index
